# Nanosilica modulates C:N:P stoichiometry attenuating phosphorus toxicity more than deficiency in *Megathyrsus maximus* cultivated in an Oxisol and Entisol

**DOI:** 10.1038/s41598-023-37504-3

**Published:** 2023-06-24

**Authors:** Cíntia Cármen de Faria Melo, Danilo Silva Amaral, Renato de Mello Prado, Anderson de Moura Zanine, Daniele de Jesus Ferreira, Marisa de Cássia Piccolo

**Affiliations:** 1 Laboratory of Plant Nutrition, Department of Agricultural Production Sciences (Soil and Fertilizer Sector), School of Agricultural and Veterinarian Sciences, São Paulo State University (UNESP), Prof. Paulo Donato Castellane Avenue, Jaboticabal, SP 14884900 Brazil; 2 Department of Engineering and Exact Sciences, School of Agricultural and Veterinarian Sciences, São Paulo State University (UNESP), Prof. Paulo Donato Castellane Avenue, Jaboticabal, SP 14884900 Brazil; 3 Center for Agricultural and Environmental Sciences, Department of Animal Science, Federal University of Maranhão, BR 222 Km 04 Highway, Chapadinha, MA 65500000 Brazil; 4 Laboratory of Nutrient Cycling, Center of Nuclear Energy in Agriculture, University of São Paulo (USP), 303 Centenário Avenue, Piracicaba, SP 13400970 Brazil

**Keywords:** Plant physiology, Abiotic

## Abstract

Silicon (Si) nanoparticles can attenuate nutritional disorders caused by phosphorus in forages through nutritional homeostasis. This paper aims to evaluate the effects of P deficiency and toxicity in *Megathyrsus maximus* cultivated in two types of soils and to verify whether Si application via fertigation can mitigate these imbalances. The following two experiments were carried out: cultivation of forage plants in pots with Entisol and Oxisol, in a 3 × 2 factorial design, with three nutritional levels of phosphorus (deficient, adequate, and excessive) and two Si concentrations in the irrigation water (0 and 1.5 mmol L^−1^). Height, number of tillers, rate of leaf senescence, dry matter production, C:N, C:Si, C:P, and N:P ratios; and C, P, and N use efficiencies were evaluated in two growth cycles. P imbalances hampered carbon assimilation, C:N:P homeostasis, and dry matter production. Nanosilica fertigation promoted silicon uptake, improving C:N:P homeostasis and nutritional efficiency in plants under P deficiency and toxicity. Leaf senescence was reduced with addition of Si in plants grown in Oxisol in the three nutritional states of P. Silicon attenuated the stress caused by P toxicity in Entisol and Oxisol, improving production in plants without nutritional stress in Oxisol. The supply of Si nanoparticles in the cultivation of *M. maximus* can contribute to a more efficient and sustainable use of phosphorus in pastures.

## Introduction

Forage plants (Poaceae) are found in different countries^1^, constituting the main feed source in cattle raised worldwide^2^. Forage cultivated in tropical soils are subjected to nutritional stress due to the limitation of nutrients in the soil (especially P), which is a fact of worldwide occurrence in pasture production^3,4^. Phosphate has low availability in soils due to strong weathering and/or sorption of the element in the soil matrix^5,6^, reducing tillering and the production of forage grasses^7^. In another scenario, intensive cultivation based on the empirical and frequent use of mineral fertilizers^8^, as well as on the frequent use of organic compounds rich in P in pastures^9^ may contribute to induce excess P in plants^10–12^.

P toxicity in forage plants cultivated in soils is still little discussed, especially in soils with a sandy texture (Entisol), which have a low P adsorption capacity compared to clayey soils, such as an Oxisol, resulting in a high concentration of the element in solution, increasing the risk of toxicity in plants^13,14^. Among the metabolic disorders caused by P toxicity, there are the reduction of enzymatic reactions^15^, chlorophyll content^16^, and photosynthetic rate^17^. This nutritional disorder is aggravated by increased N uptake^18^, which unbalances C:N and C:P homeostasis^19^ and can accelerate leaf senescence, causing leaf chlorosis and/or necrosis^20,21^.

A sustainable strategy to minimize the effects of P imbalance in forage would be the use of silicon (Si), as forage are plants that accumulate this element^22^ and this is a practice that does not pose a risk to the environment. Extensive reviews indicate that Si provides plant resilience under stressful conditions^23–26^, but studies involving Si mitigating P stress in forages are limited. The absorption of Si by plants occurs in the form of monosilicic acid (H_4_SiO_4_)^27,28^, which is transported by the apoplast and symplast to the root xylem. When it reaches the leaves, it is mainly deposited on the cell walls, being polymerized, forming phytoliths and silica bodies^29^.

Tropical soils extensively cultivated with pastures present a high total Si content, but in unavailable forms^30^. The Si concentration available in the soil solution is relatively low, ranging from 0.1 to 0.6 mM L^−1^ of Si, which is caused by the intense weathering in these soils, added to the successive extraction of Si from crops without carrying out replacement, causing desilication^31,32^, and the successive extraction of Si from crops without fertilization for tis replacement. Given the above context, it is important to supply Si for crops with low solubility sources, such as steel slags in the form of solid calcium silicate, which are incorporated into the soil^33,34^. However, the application of Si in forages (through sodium and potassium silicate) has been shown to be more efficient through fertigation^35–38^, and recently, with the advancement of nanotechnology, Si nanoparticles have emerged^39,40^. Nanosilica has a nanoporous structure, with particle diameters of up to 100 nm and high specific surface area^41^, which can favor absorption and enhance the accumulation of Si in the plant shoot. The use of nanosilica should be studied especially in Si-accumulating crops, such as forages plants.

A new approach discusses the benefits of Si for nutrient uptake in plants under limiting conditions^42^ and for the modification of C:N:P stoichiometry in grasses, attenuating the water deficit^38,43^ and nutritional deficiency in forage plants under hydroponic cultivation^44^. This change in C stoichiometry must be related to the incorporation of Si into the cell wall, being metabolized at a low energy cost compared to the synthesis of structural carbon compounds such as lignin^45,46^, and these factors may favor plant growth under stress. Silicon can increase P uptake in P-deficient sorghum plants^47^, and also interact with the soil, occupying phosphate adsorption sites, increasing the availability of P in the soil^42^. In rice plants under excess P, the beneficial element decreases P uptake by downregulating the P transporter gene^48^, which may be a plant strategy for the homeostasis of this macronutrient in plant tissues.

Nutritional disorders related to P can cause disturbances in C and N homeostasis, aggravating its efficient nutritional use by forage plants, but there is a lack of research to prove this. The same applies to the potential of Si to reverse these stresses. Thus, in order to advance in the present topic, some hypotheses need to be tested: (i) whether the damage to the growth of forage plants under P deficiency and to plants under P excess is related to the modification of C:N:P stoichiometry, consequently causing the loss of nutritional homeostasis and decreasing the efficiency of use of C, N and P by the plant; (ii) whether the risk of P toxicity in forage plants is greater in soils with a sandy texture compared to clayey textures; (iii) and whether the application of Si in the form of a nanosilica-based fluid to the soil promotes the optimal absorption of the element for the plant, being sufficient to attenuate the deficiency of P or excess P through the C:N:P stoichiometric homeostasis, and the increase in efficiency of use of these nutrients in the plant depending on the cultivated soil.

If these hypotheses are confirmed, the underlying mode of action of Si on the stoichiometric homeostasis of forage plants cultivated in different soils under nutritional stress due to P deficiency or excess P will be verified. Therefore, the potential of nanosilica will be verified for the first time as a strategy to maximize the growth of forages in different cropping systems, whether in low-tech cultivation systems with sub-doses of P or in intensive cultivation, with the use of high doses of P and problems of excess in plants. It will still be known if the beneficial effect of Si in the attenuation of P imbalances may or may not vary according to the soil class. Thus, the optimized management of Si should boost the production of sustainable forage without risks to the environment.

In this context, this study was carried out with the objective of evaluating whether P imbalances alter the C:N:P stoichiometry in the *Megathyrsus maximus*, whether Si applied to the soil as nanoparticles attenuates the effects of P deficiency and toxicity, and improves C:N:P homeostasis by optimizing plant growth under P phosphorus levels (deficient, adequate and excessive) in Oxisol and Entisol.

## Material and methods

### Location and experimental design

Two independent experiments were carried out at the São Paulo State University (UNESP), municipality of Jaboticabal, Brazil, in 2021, under greenhouse conditions (one experiment was conducted in an Entisol (Quartzipsamment), and another experiment was conducted in an Oxisol)^49^, with the forage plant *M. maximus* cv. Zuri. We declare that the experiments were carried out in accordance with relevant guidelines and regulations. Treatments were arranged in a 3 × 2 factorial design, with three P nutritional levels of: deficient (zero application of P), adequate or sufficient (200 mg dm^−3^ of P), and excessive (600 mg dm^−3^ of P)^58^, combined with the absence of Si (zero Si) and the presence of Si (1.5 mM), with four replicates, in a completely randomized design. The experimental unit consisted of two forage plants in a plastic pot (height: 32 cm; lower and upper base: 15 cm) filled with 6 kg of Oxisol sample and 8 kg of Entisol sample. The plants were cultivated during two growth cycles, that is, two cuts were performed in a total experimental period of approximately 90 days from sowing.

### Installation of the experiments

For both experiments, soils were collected from the surface layer of the uncultivated area, in the 0–20 cm layer. The samples were air-dried and passed through a 6-mm mesh sieve, being subsequently subjected to chemical analysis (for soil fertility)^50^ and Si content analysis^51^. The results of the chemical analysis for the Oxisol and Entisol were, respectively: pH in CaCl2 = 3.8 and 4.3; OM (organic matter) = 34 and 9.0 mg dm^−3^; P in resin extractor = 12 and 2 mg dm^−3^; K = 1.4 and 3.0 mmolc dm^−3^; Ca = 6 and 3.0 mmolc dm^−3^; Mg = 2 and 1.0 mmolc dm^−3^; H + Al = 85 and 16; T (cation exchange capacity) = 94 and 20.3 mmolc dm^−3^; V (base saturation): = 10 and 21%; and Si = 3.0 and 1.0 mg dm^−3^. The granulometric analysis carried out according to^52^ showed the following results for Oxisol and Entisol, respectively: 51 and 94% sand, 6 and 1% silt, and 43 and 5% clay.

Limestone (total neutralization power: 125%, CaO: 58.5%, MgO: 9%) was applied to the soils to correct acidity and to increase base saturation to 70%^50^, corresponding to doses of 0.48 and 2.25 g dm^−3^ in the Entisol and Oxisol, respectively. The limestone was homogenized with the soil, and soils were maintained moist and incubated for 40 days before sowing.

Fertilization was carried out through soil application by supplying K, S, Zn, and B at doses of 150, 50, 5, and 0.5 mg dm^−3^, respectively, besides supplying 5 mg dm^−3^ of Fe to the Entisol, using the following sources: potassium chloride, calcium sulfate, zinc sulfate, boric acid, and iron chelate. Phosphorus was applied at doses of 0, 200^53^, and 600 mg dm^−3^ in the plots corresponding to the deficient, adequate, and excessive levels of P, respectively, in the form of triple superphosphate. Nitrogen fertilization was carried out by N topdressing with 300 mg dm^−3^ of N in installments during each regrowth period, being always incorporated with irrigation.

Sowing was carried out directly in the soil of each plot by manually depositing approximately 20 seeds of *M. maximus* cv. Zuri in a circular groove at a depth of 1 cm. After seedling emergence, thinning was performed, maintaining two plants per pot. At 30 days after sowing, a uniform cut was carried out at 17 cm from the ground level in all pots to stimulate tillering, initiating the splitting of nitrogen fertilization and Si application, as well as the period of data collection. On that occasion, 4 mg dm^−3^ of P were applied to the plants in the deficient treatment (Entisol) to enable the minimum growth of these plants.

### Experimental conditions

The temperature and relative humidity inside the greenhouse were monitored daily using a thermo-hygrometer. The maximum average temperature recorded was equal to 46.3 ± 5 °C, with a minimum average temperature of 21.8 ± 3 °C and average relative humidity equal to 47.6 ± 10%.

The water-holding capacity or available water capacity (AWC) of the soils was determined. Two pots filled with each soil were subjected to saturation in a container with water for 24 h. After saturation, the pots were covered with plastic and removed from the saturated environment for free drainage. After draining, soil samples were dried in an oven and the AWC was determined by the difference between the masses of drained (wet) and dry soil. The management of water replacement was established by maintaining the soils at 70% of AWC, a condition indicated for Poaceae^54^, under which water is available and gas exchanges are maintained in the root zone. The pots were weighed daily in the late afternoon, after which evapotranspirated water was replaced^55^.

### Silicon supply

Silicon was supplied daily from the moment of sowing until the last cut of the plants, using a solution of 1.5 mmol L^−1^ of Si^36^. The solution was prepared with colloidal nanosilica (particle size between 8.5 and 9.7 nm, specific surface area of 300 m^2^ g^−1^, and pH 10.5). The same volume of solution was applied in all treatments, with the volume being defined based on the treatment with the smallest daily water demand. Thus, in treatments with greater water demand, irrigation was supplemented with deionized water. The volume of solution applied was quantified at the end of the experimental period, totaling 26.2 and 22.3 L of solution applied per pot, providing 1.09 and 0.94 g per pot of Si in the Oxisol and Entisol, respectively. In plots that did not receive Si, irrigation was always performed with distilled and deionized water.

### Analysis

#### Height, tillering, and senescence of plants

At the end of each of the two regrowth cycles, plant height (cm) was measured considering the length from the base to the inflection of the fully developed leaves, using a ruler. The number of tillers, as well as green and senescent leaves were counted to calculate the percentage of senescent leaves per pot (%).

#### Biomass production

The plants were cut twice when reaching 70 cm in height in the treatment with adequate P content^56^. This height was reached at 25 and 31 days after the uniform cut in the Entisol and Oxisol, respectively, when the first cut was made. At 28 and 32 days of regrowth in the Entisol and Oxisol, respectively, the second cut was performed. The cut was performed using scissors, leaving 30 cm of residual material above the ground and collecting only the mass of the grazing strata of plants. Then, samples were washed in detergent solution (0.1% v:v) and deionized water, successively, being dried in an oven with forced air circulation (65 ± 5 ºC) until reaching constant mass. After reaching constant mass, shoot dry matter per plot was determined (DM, g per pot). The dried material was then passed through a Willey mill.

#### Contents of C, N, P, and Si

C content was determined through wet digestion of the dry matter with a K_2_Cr_2_O_7_ solution and titration with FeSO_4_ by the modified Walkley–Black method^57^. To evaluate N content, the sample was submitted to digestion with sulfuric acid, distillation by the Kjeldahl method, and determination by titration^58^. P was determined from nitric-perchloric acid digestion and reading in a spectrophotometer at 420 nm^59^. Si was extracted with alkaline digestion in hydrogen peroxide at 120 °C^60^ and determined by reading in a spectrophotometer at 410 nm after a colorimetric reaction with ammonium molybdate^51^. The contents of each element were expressed in g kg^−1^.

#### Stoichiometric ratios and use efficiency

C:N, C:P, N:P, and C:Si stoichiometric ratios were calculated using the contents of the elements in the dry matter. The use efficiencies of C, N, and P were calculated using the expression: ((total dry matter produced)^2^/(total accumulation of nutrient in the plant))^61^.

### Statistical analysis

The assumptions of the analysis of variance were verified by testing normality, homogeneity, independence of residuals^62,63^, and the presence of outliers^64^. The data from each experiment were submitted to analysis of variance using the F test (*p* < 0.05). Means were compared using the Student–Newman–Keuls (SNK) test at 5% probability, using the statistical software SPEED Stat, version 2.4^65^.

## Results

### P, Si, C, and N contents

**Figure 1:**
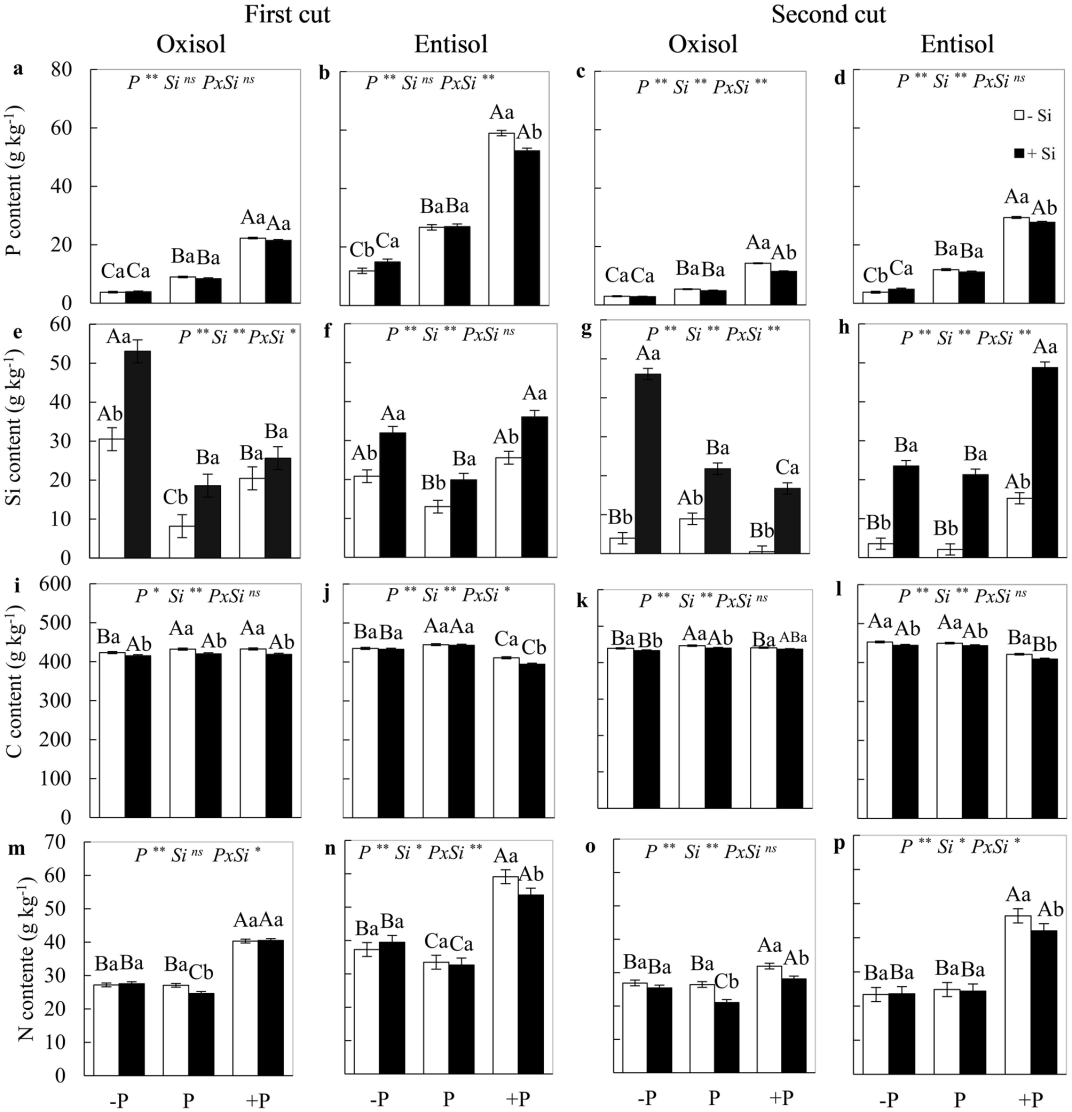
Phosphorous, silicon, carbon, and nitrogen content in the dry mass of the forage plant *M. maximus* cv. BRS Zuri, in the first (**a,b,e,f,i,j,m,n**) and second cuts (**c,d,g,h,k,l,o,p**), cultivated under phosphorus levels of deficiency (− P), adequacy (P), and excess (+ P) in Oxisol and Entisol, in the absence (− Si) and presence (+ Si, 1.5 mmol L^−1^) of silicon. Ns, * and ** correspond to the non-significant F-test, significant at 5 and 1%, respectively. Lowercase letters indicate differences from Si within each phosphorus level, while uppercase letters indicate phosphorus levels according to the Si condition (SNK test, 5% probability). Bars represent the standard error of the mean, n = 4.

In most situations evaluated (cuts and soils) there was a significant P x Si interaction (*p* < 0.05) in the P, Si, C and N contents, that is, Si influences these contents differently depending on the state of P. Similarly, the effect of P state can also be influenced by the absence or presence of Si. Plants cultivated in the condition of P deficiency and excess P in the absence of Si showed lower and higher levels of the element in the plant, respectively, in both soils and forage cuts (Fig. 1a–d). In the absence of Si, the nutritional status of P influenced the Si content in the plants, resulting in a higher Si content in plants under P deficiency and excess P in Entisol in the first cut and under excess P in the second cut, as well as in plants under P deficiency of P in Oxisol in the first cut (Fig. 1e–h). The C content in plants grown without Si application was lower under P deficiency in both cuts and under excess P in the second cut in Oxisol (Fig. 1i,k). In the cultivation in Entisol in the absence of Si, a lower C content was observed in treatments with excess P in both cuts, as well as under deficiency in the first cut (Fig. 1j,l). In the absence of Si, there was higher N content in the plant under excess P in both cuts and soils (Fig. 1m–p).

In the presence of Si in Oxisol, a reduction in P content was observed in the condition of excess P in the second cut (Fig. 1c), while in plants cultivated in Entisol there was a higher P content in P-deficient plants and a lower P content in P-excess plants, as well as a lower P content in plants with excess P in both cuts (Fig. 1b,d). The application of Si in relation to its absence promoted a higher Si content in the plant for all nutritional levels of P, soils, and forage cuts (Fig. 1e–h). The C content of plants was lower in the presence of Si for all nutritional levels of P in the first cut in Oxisol and in the second cut in Entisol, as well as under excess P in the first cut in Entisol and in the deficiency and sufficiency of P in the second cut in Oxisol (Fig. 1i–l). In the presence of Si, there was a lower N content in plants under P sufficiency in both cuts and under excess P in the second cut in Oxisol (Fig. 1m,o), as well as in plants under excess P grown in Entisol in both cuts (Fig. 1n,p).

### C:Si, C:N, C:P and N:P stoichiometric ratios

**Figure 2:**
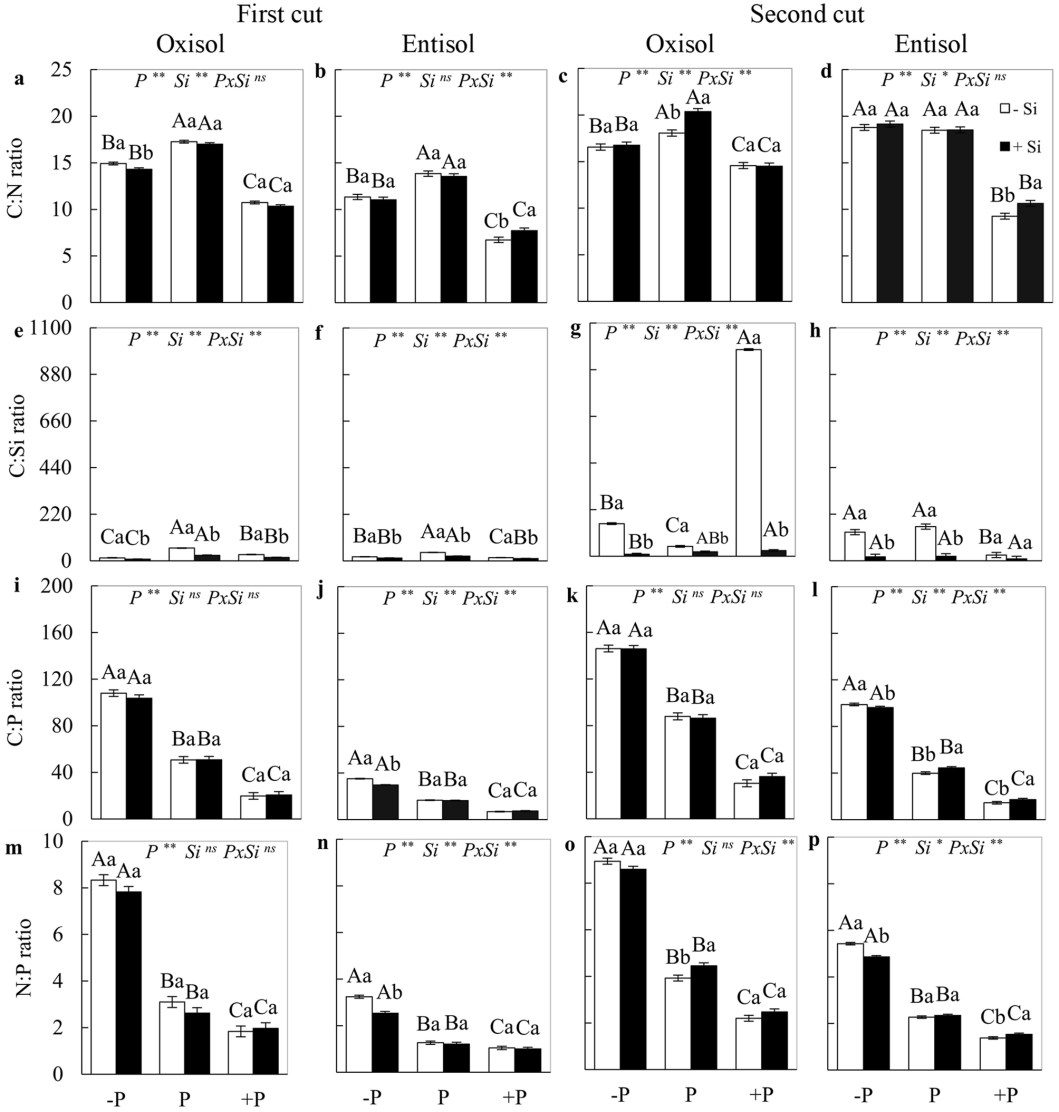
C:N, C:Si, C:P and N:P stoichiometric ratios in the forage plant *M. maximus* cv. Zuri, in the first (**a,b,e,f,i,j,m,n**) and second cuts (**c,d,g,h,k,l,o,p**), cultivated under phosphorus levels of deficiency (− P ), adequacy (P), and excess (+ P) in Oxisol and Entisol, in the absence (–Si) and presence (+ Si 1.5 mmol L^−1^) of silicon. Ns, * and ** correspond to the non-significant F-test, significant at 5 and 1%, respectively. Lowercase letters indicate differences from Si within each phosphorus level, while uppercase letters indicate phosphorus levels within each Si condition (SNK test, 5% probability). Bars represent the standard error of the mean, n = 4.

The P *x* Si interaction was significant (*p* < 0.05) for the C:Si ratio in both soils and cuts, and in at least one cut in each soil type for the C:N, C:P and N:P ratios. Cultivation with the absence of Si resulted in the lowest value of the C:N ratio in plants under P deficiency and excess P in both soils, with the exception of the deficiency in the second cut in Entisol, in which the C:N ratio was not affected (Fig. 2a–d). The absence of Si in forage plant cultivation also resulted in higher C:N and C:Si ratios in the adequate nutritional status of P in both soils, decreasing in plants with P imbalances, except for the second cut of plants under excess P in Oxisol and under P deficiency in Entisol (Fig. 2a–h). The highest C:P and N:P ratios occurred in plants under deficiency, while the lowest value of these ratios occurred in plants with excess P, respectively, in both cuts and soils (Fig. 2i–l; m–p).

Fertigation with Si provided a lower C:Si ratio for plants, regardless of soil type or cut (Fig. 2e–h). In plants grown in Entisol, the presence of Si resulted in the lowest C:P and N:P ratios under P deficiency in both cuts, while resulting in the highest value of these stoichiometric ratios in plants grown under excess P in the second cut (Fig. 2j,l,n,p). In plants cultivated in Oxisol, the presence of Si resulted in an increase in the N:P ratio under the adequate level of P in the second cut of the grass forage (Fig. 2i,k,m,o).

### Use efficiency of P, C, and N

**Figure 3:**
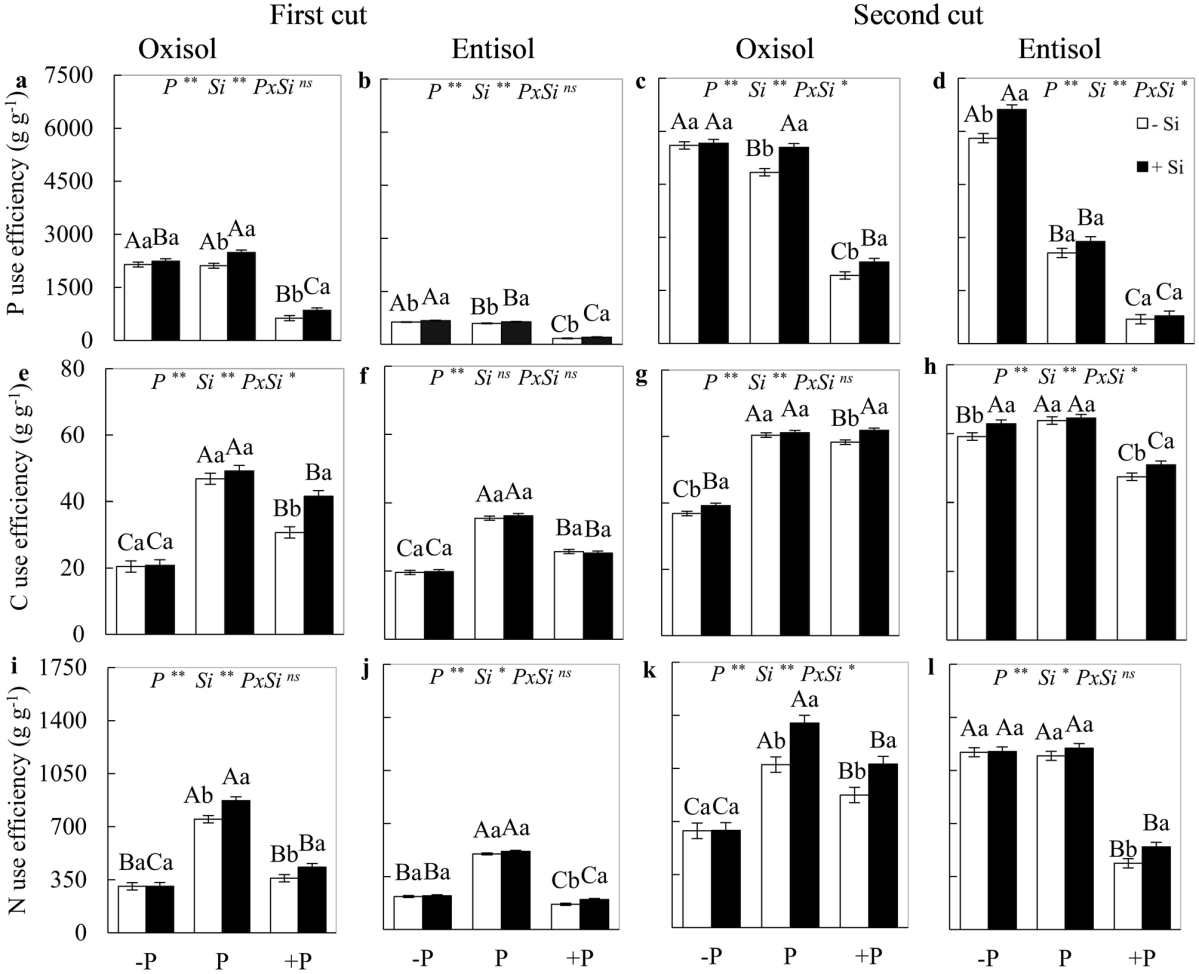
Use efficiencies of phosphorus, carbon, and nitrogen in the forage plant *M. maximus* cv. BRS Zuri, in the first (**a,b,e,f,i,j**) and second cuts (**c,d,g,h,k,l**), cultivated under phosphorus levels of deficiency (− P), adequacy (P), and excess (P) in Oxisol and Entisol, in the absence (− Si) and presence (+ Si, 1.5 mmol L^−1^) of silicon. Ns, * and ** correspond to the non-significant F-test, significant at 5 and 1%, respectively. Lowercase letters indicate differences from Si within each phosphorus level, while uppercase letters indicate phosphorus levels within each Si condition (SNK test, 5% probability). Bars represent the standard error of the mean, n = 4.

The P *x* Si interaction was significant (*p* < 0.05) for P use efficiency in the second cut in both soils. In the absence of Si fertigation, P use efficiency by the plants was higher under nutrient deficiency in both cuts and soils, except for the first cut in Oxisol (Fig. 3a–d). P deficiency and excess P in the forage grass resulted in lower C and N use efficiency by the plants in both soils and cuts, with the exception of P deficiency in Entisol in the second cut, in which N use efficiency was not affected (Fig. 3e–l).

When there was the addition of Si, a greater value for P use efficiency was observed in plants under the three nutritional levels of P in the first cut, as well as in plants under P deficiency in the second cut and cultivated in Entisol (Fig. 3b,d). In plants grown in Oxisol, the presence of Si resulted in a higher P use efficiency only under the adequate and excessive levels of P in both cuts (Fig. 3a,c), while higher C use efficiency was observed in plants under P deficiency in the second cut (Fig. 3g) and in the condition of excess P in both forage cuts (Fig. 3e,g). In plants grown in Entisol, fertigation with Si resulted in the highest C use efficiency in plants under both P imbalances, being restricted to the second cut (Fig. 3h). There was an increase in N use efficiency in plants grown with Si in adequate nutritional status and excess P grown in Oxisol (Fig. 3i,k), as well as in plants with excess P grown in Entisol in both cuts (Fig. 3j,l). There was no effect of Si on N use efficiency of P-deficient plants in any of the evaluated soils and cuts (Fig. 3i–l).

### Height, tillering, senescence, and dry matter production

**Figure 4:**
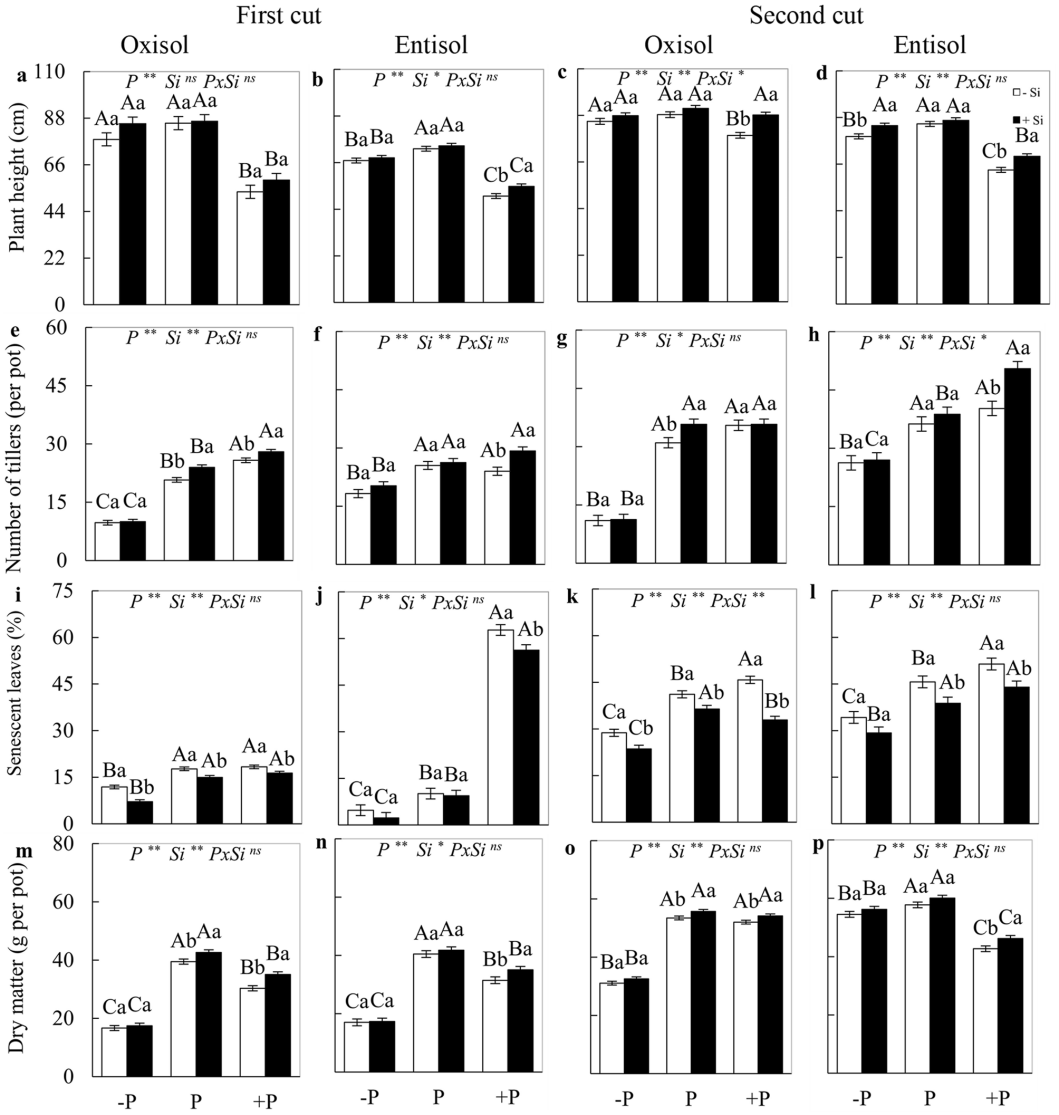
Plant height, number of tillers, rate of leaf senescence, and biomass production in the grazing strata, in the first (**a,b,e,f,i,j,m,n**) and second cuts (**c,d,g,h,k,l,o,p**), in the forage plant *M. maximus* cv. BRS Zuri cultivated under phosphorus levels of deficiency (− P), adequacy (P), and excess (+ P) in Oxisol and Entisol, in the absence (− Si) and presence (+ Si, 1.5 mmol L^−1^) of silicon. Ns, * and ** correspond to the non-significant F-test, significant at 5 and 1%, respectively. Means were tested by the SNK test at 5% error probability. Uppercase letters compare means between nutritional levels and lowercase letters compare the effect of Si. Bars represent the standard error of the mean, n = 4.

For the parameters plant height and percentage of senescent leaves, there was a significant P *x* Si interaction (*p* < 0.05) in the second cut in Oxisol, and also for the number of tillers in Entisol, while for the dry matter production the factors acted independently. In the absence of Si, plant height presented a lower value in the nutritional condition of excess P in both soils and cuts, as well as in Entisol under P deficiency in both cuts in relation to plants with adequate P status (Fig. 4a–d). In the absence of Si, the lowest value for the number of tillers occurred in plants under P deficiency in both soils and cuts (Fig. 4e–h), while the highest value occurred in plants under excess P grown in Oxisol in the first cut (Fig. 4e). Excess P resulted in the highest rate of foliar senescence, except for the first cut in Oxisol (Fig. 4i–l).

Shoot dry matter production in the absence of Si showed the lowest value in P-deficient plants grown in Oxisol in both cuts (Fig. 4m,o), as well as in plants grown in Entisol in the first cut (Fig. 4n). In plants under excess P and under the absence of Si, the lowest dry matter production occurred in Entisol and in both cuts (Fig. 4n,p). However, when plants were cultivated in Oxisol, this occurred only in the first cut (Fig. 4m).

Under excess P, plants fertigated with Si showed an increase in height in both soils and cuts, except for the first cut in Oxisol (Fig. 4a–d). In plants grown in Oxisol, there was a greater number of tillers in the adequate level of P in both cuts (Fig. 4e,g), as well as under excess P in the first cut in relation to the absence of Si (Fig. 4e). In plants grown in Entisol in the presence of Si, there was a greater number of tillers in plants under excess P in both cuts (Fig. 4f,h).

Under excess P, plants fertigated with Si showed an increase in height in both soils and cuts, except for the first cut in Oxisol (Fig. 4a–d). In plants grown in Oxisol, there was a higher number of tillers under the adequate level of P in both cuts (Fig. 4e,g), as well as under excess P in the first cut in relation to the absence of Si (Fig. 4e). In plants cultivated in Entisol and in the presence of Si, there was a greater number of tillers in plants under excess P in both cuts (Fig. 4f,h).

The rate of leaf senescence decreased with the addition of Si in relation to its absence in plants grown in Oxisol in the three nutritional levels of P and both cuts (Fig. 4i,k). In plants cultivated in Entisol, the rate of leaf senescence decreased with the addition of Si in plants under excess P in both cuts, as well as in the adequate level of P in the second forage cut (Fig. 4j,l). Si supply increased the dry matter production of plants under excess P grown in Entisol in both cuts (Fig. 4n,p), as well as in plants grown under adequate and excessive levels of P in Oxissol, in both cuts (Fig. 4m,o), with no effect on macronutrient deficiency in any of the soils or cuts.

## Discussion

### Biological damage caused by P imbalances in the plant without Si supply

The deficiency of P in the forage was clear, as plants cultivated without P application showed low P levels both in the plant cultivated in Oxisol (3.4 g kg^−1^) and in Entisol (7.8 g kg^−1^) in relation to the plant grown under the adequate P level in Oxisol (7.2 g kg^−1)^ and Entisol (19.1 g kg^−1^). P deficiency in the plant was expected, as the soils used in this experiment presented low levels of available P. This is common in weathered soils, especially in Oxisol, which has a high phosphate adsorption capacity^66,67^.

Studies on the deficiency of P in forage plants are restricted to the evaluation of nutrient levels and symptoms^68–70^, not analyzing whether this disorder causes stoichiometric changes. Thus, for the first time in a forage plant, it was verified that P deficiency decreased the C:Si ratio in plants, especially in the first cut, in plants grown in sandy or clayey soil. This is because plants deficient in P presented a higher Si content compared to those under sufficiency due to greater Si absorption even without application of the element. This result confirms that the forage plant develops the ability to absorb natural Si from the soil when subjected to nutritional stress^24^. It is possible that the increase in Si absorption may have been favored by the liming of the soil, as phytoliths (a material rich in Si previously deposited in the soil) can be dissolved through an increase in the pH of the solution^71^, favoring the availability of the element in the soil. However, in the second cycle there was a decrease in Si content in these P-deficient plants compared to the first forage cycle, confirming that although phytoliths are a source of Si^72,73^, they do not sustain Si uptake over successive cuts.

P deficiency by decreasing the C content of the plants compared to those under sufficient or adequate levels of P reflected in a decrease in the C:N ratio of the forage in both soils and cuts, except for the second cut in plants grown in Entisol. The decrease in C content in P-deficient plants observed in this study is similar to that reported for another species (*Holcus lanatus*)^74^, which confirms the essential role played by P in the assimilation of C, as it regulates photosynthesis by acting as substrate, product, and/or effector of enzymes of the C4 pathway in mesophyll cells^68,75^. Thus, even with a possible recycling of P in deficient plants^8^, it is not enough to meet the demand of the plant organs generating nutrient deficiency, and its damage is well documented in photosynthetic efficiency^47^.

Changes in C:N:P stoichiometric ratios in deficient plants resulted in greater P use efficiency compared to plants grown under P sufficiency. This is natural in plants grown in soils with restricted P contents due to mechanisms such as membrane remodeling with P-free lipids (such as galacto- and sulfolipids)^70^ or due to the preservation of P in inorganic forms, facilitating its remobilization^76^. However, there was a decrease in the use efficiencies of N and C because the conversion of N and C into biomass depends on P cellular homeostasis, as it is essential to the physiological and biochemical processes in plant cells, being a component of nucleic acids and membrane lipids, as well as a mediator of the energy metabolism^77,78^.

The efficiency of plants to use absorbed nutrients to compose its tissues has a direct influence on biomass production^61,79^. Therefore, P deficiency in relation to P-sufficient plants reduced nutritional homeostasis and the use efficiency of C and N, affecting forage plant growth in both cycles and in the two soils studied, with a decrease in dry matter production dries of 50 and 31% in plants grown in Oxisol and Entisol, respectively. These losses have been commonly reported in Poaceae cultivated under low levels of P^44,80^, limiting the productive capacity of pastures in tropical soils^3,4^.

Another important nutritional disorder in plants is P toxicity. It is still little reported in forage grasses, especially in crops without silicate fertilization, which is the condition of most pastures in the world. In this research, it was clearly evident that the high dose of P applied reflected in high average levels of P in the plant, reaching approximately 18.3 and 44.2 g kg^−1^ compared to the treatment under P sufficiency, which presented 7.2 and 19.1 g kg^−1^ of P in plants grown in Oxisol and Entisol, respectively. It should be noted that the lower P content in plants grown in Oxisol compared to Entisol is probably due to the greater adsorption of the element in Oxisol, reducing P availability in the soil and its absorption by the plant^80^. The opposite occurred in Entisol, which provided high levels of P in the plant due to the low clay content in this soil, giving it a low adsorption capacity for phosphate and greater availability for the plant, with a greater risk of toxicity in the plants^81,82^.

It is worth mentioning that the stress resulting from excess P in the plant stimulated the absorption of residual Si from the soil by the forage plant (as it occurred under P deficiency). This confirms that the plant uses the strategy of absorbing Si even at low levels in the soil, which may be a plant defense mechanism to mitigate stresses^24,83^.

An aspect not yet reported in forage plants is the effect of toxicity or excess P on C:N:P stoichiometry. It was evidenced in this research that the excess P in relation to the sufficiency of P affected the C:N:P stoichiometric ratios in the forage, with a decrease in the C:P, N:P , and C:N ratios in the two studied soils. The C:N ratio is highlighted for decreasing on average 28 and 49% in plants grown in Oxisol and Entisol, respectively. This reduction was due to the combination of a lower C content and a higher N content in plants under excess P, inhibiting the activation of RubBisCO by decreasing photosynthesis^15,84^.

In addition, we also showed that lower C:P and N:P ratios may indicate that C and N can limit P toxicity in the plant. Although there was a greater N uptake, the generated stoichiometric imbalance limits the use of nutrients^85^. Thus, the loss of C:N:P homeostasis had nutritional consequences on the plant, as it decreased the use efficiencies of P, C, and N in plants grown in both soils and cuts because the conversion of nutrients into biomass depends on the possibility and/or capacity of the plant to maintain sufficient concentrations while maintaining in stoichiometric equilibrium in the tissues^19,86^.

As a result of the decrease in nutrient use efficiencies, excess P reduced plant growth, decreasing plant height and increasing the rate of leaf senescence in plants cultivated in both soils, thus causing a 24% decrease in dry matter production in the two soils and two cuts of forage cultivated in Entisol, while causing a 23% decrease in the first cycle of plants cultivated in Oxisol. Thus, the physiological damage of P toxicity (especially in photosynthesis)^17,84^ was previously induced by nutritional disturbances in elemental homeostasis.

**Figure 5:**
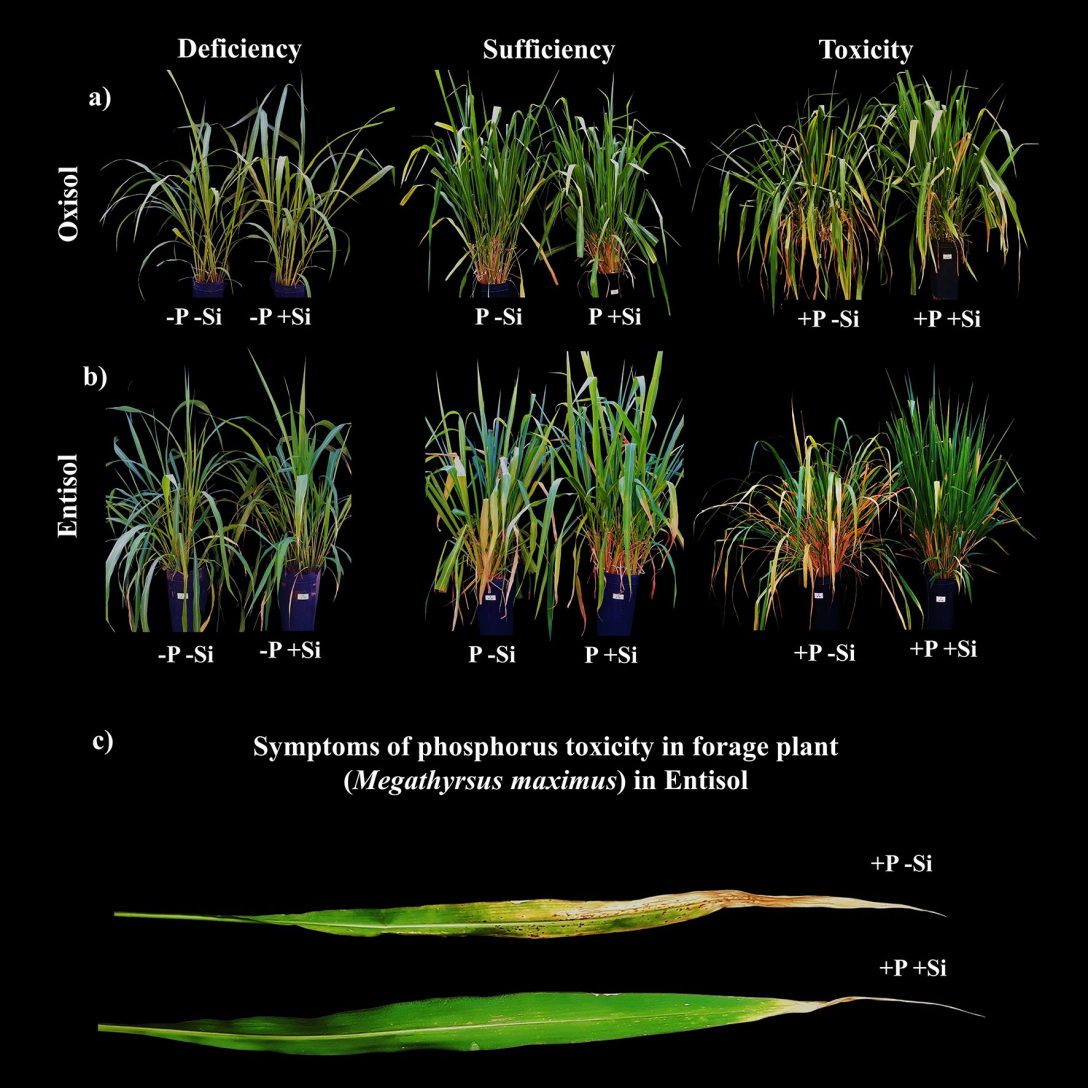
Visual aspects of *M. maximus* cv. Zuri under phosphorus deficiency (− P), sufficiency (P), and toxicity (+ P) combined with the absence (− Si) and presence of Si (+ Si) nanoparticles grown in Oxisol (**a**) and Entisol (**b**); details of symptoms of P toxicity in *M. maximus* in the absence and presence of Si (**c**).

Reflections of P imbalance were observed for the first time in the species *M. maximus*. P toxicity begins with an excess of thin tillers and leaves with reduced length, not very rigid and decumbent (Fig. 5a,b). In addition, there was chlorosis in older leaves, with purplish spots that progress to necrosis from the apex to the base in the leaf blade (Fig. 5c), bein similar to what was described in leaves of wheat and rice plants under P toxicity^84,87^.

Thus, the observed results allow accepting the first and second hypotheses tested, as it was shown that P imbalances modify the C:N:P stoichiometry of the forage and that P toxicity caused a greater stoichiometric imbalance especially in plants cultivated in a sandy soil (Entisol). Therefore, the risk of facing P toxicity is high for forages grown in Entisol, requiring a rigorous management of this nutrient.

### Benefits of Si in mitigating stress under P deficiency and toxicity in grass forage

Si is a well-known element to attenuate different abiotic stresses in many crops. However, in forage plants, studies evaluating P deficiency were carried out only in hydroponic cultivation^36,44^, and there is a lack of reports of crops in different soils, especially regarding P toxicity. This is worrying, as forage crops are grown in different soils worldwide, with vast areas cultivated in Oxisol and Entisol. Thus, it is important to know if Si is really capable of mitigating these stresses under these cultivation conditions.

Initially, it was evident that plants cultivated under P deficiency in Entisol and that received the application of Si nanoparticles presented an increase of 21% in the P content in relation to those that did not receive Si. It is important to note that the levels of Si in the plant were higher in deficient plants than in plants under P sufficiency as a in response to stress. Therefore, a higher Si content in the soil, and consequently in the plant, has the ability to positively regulate the gene expression of P transporters, in addition to favoring the availability of P in the soil by stimulating the exudation of malate and citrate in Poaceae^88^, decreasing the adsorption and favoring the uptake of P by the plant. In this context, the use of Si can favor a greater use of the P present in the soil by pastures, which is essential, since nutrient reserves are finite^89^ 25% of the land cultivated with pastures has soils with low P content^1,4^.

Additionally, Si promoted a decrease in C:P and N:P ratios in the two cycles in Entisol, thus promoting a 9% increase in the P use efficiency by the plant. This was also reflected in greater C use efficiency in the second forage cut in both soils. Complementarily, we carried out a Pearson correlation analysis between the studied variables and the DM production of the deficient plants, and we observed that the use efficiencies of C (r = 0.996^*^) and P (r = 0.991^*^), as well as the C:P ratio (r = 0.986^*^) were the ones that most contributed to DM production in both soils. A similar action was observed in rice plants deficient in N, in which Si promoted greater nutritional homeostasis, contributing to the efficient use of nutrients^35^. The benefits of Si in the balance of C:N:P stoichiometry were not enough to increase the efficiency of N use in the forage in the two cuts and in the two soils. Consequently, there was no increase in the number of tillers or in dry matter production by plants deficient in P. However, other authors have observed this effect of Si in other pastures^46^ and in sorghum^47^. It is possible that the effect of Si on C:N:P homeostasis did not reach a sufficient level to favor greater accumulation of biomass and mitigate P deficiency in the forage within the growth cycle studied. Considering the potential of Si presented in our study, we reinforce the need for further research aimed at studying the mitigating action of Si on P-deficiency stress in Poaceae plants.

It was verified that P toxicity was severe in plants that did not receive Si, showing a limiting nutritional disorder. In this research, we observed that in plants cultivated under excess P, the increase in the absorption of the Si applied to the soil in the form of nanosilica reduced the P content in the two cuts of the forage cultivated in Entisol, as well as in the second cut in Oxisol, revealing that Si negatively modulates P absorption in conditions of excess. One of the possible ways to attenuate P toxicity by Si is the modulation of the gene expression of P transporters^90^, which has already been demonstrated in rice^8^ and depends on the translocation and accumulation of Si in the shoot^91^. Forages have a good capacity for Si absorption, as they are a Si-accumulating plants^29^, especially when the element is supplied in the form of nanosílica^92^.

The increase in Si absorption in relation to its absence in P-deficient plants caused changes in stoichiometric ratios in the forage, especially in the second cut of the plant cultivated in Entisol, with lower C:P and N:P ratios, which is due to the greater P uptake. Furthermore, Si increased the C:N ratio in the forage in both cuts in Entisol, improving stoichiometric homeostasis. Therefore, for the first time there is a report on the potential of Si to modify C:N:P stoichiometry in forage plants cultivated under excess P, but this depends on the soil used for cultivated, as it stood out more in Entisol, probably due to the greater severity of macronutrient toxicity. Research on the effects of Si on C:N:P stoichiometry in stressed plants^93^, of water deficit in sugarcane^43,55,94^, and of the effect of Si in the forage plant *M. maximus* are recent^38^.

The effects of Si in relation to its absence on the C:N:P homeostasis of forage plants cultivated under P toxicity was sufficient to increase nutritional efficiency, highlighting the efficiency of N use, providing an average increase of 18% in this efficiency, in the two cycles and soils. There was also an increase in the use efficiency of C and P depending on the forage cut. These added effects on nutritional efficiency were sufficient to favor forage growth under excess P by increasing height and number of tillers in both soils, consequently increasing forage dry matter production. In Pearson's correlation analysis, we observed that the use efficiencies of C (r = 0.996^*^), P (r = 0.979^*^) and N (r = 0.945^*^) in Entisol, and the efficiencies use of C (0.996^*^), and N (r = 0.949^*^) and plant height (r = 0.901^*^) in Oxisol, were the ones that most contributed to the increase in DM in plants under P toxicity.

An important aspect favored by Si to attenuate P toxicity and plant growth was its strong and constant effect of decreasing the rate of leaf senescence in all soils and growth cycles. It is possible that this effect of Si on leaf senescence may be related to the increase in N use efficiency, as this nutrient increases the period when leaves are photosynthetically active^78^ and also deletes genes related to mechanisms for the induction of senescence^95^. This effect of Si in reducing leaf senescence is little studied, being only reported in plants cultivated under water deficit, such as rice^96^ and sorghum^97^, and under sulfur deficiency, such as in barley plants^95^. In this paper, this effect was visually noticed (Fig. 5a,b,c).

It was also possible to visually notice the benefits of Si in mitigating P toxicity, since the damage caused by this previously described nutritional disorder was not observed when the plants received Si (Fig. 5), which presented better plant architecture, as already observed in rice plants^98^ due to greater rigidity in the plant tissues^73^.

In parallel, another benefit of Si in the plant would be its effect of increasing the energy in plant metabolism, which would favor the antioxidant defense mechanisms of plants due to P toxicity, providing components for the Poaceae cell wall, ensuring structural resistance, and reducing the need for lignin synthesis (which demands a high energy content)^99,100^. This energy can be used in the organic synthesis of plant defense compounds against stress.

Our results indicate that forages are sensitive to P toxicity, causing an imbalance of C:N:P stoichiometry in the plant, and that the use of nanosilica via fertigation can mitigate this stress, making the pasture productive again and providing environmental implications. Excess P in the soil is at risk of contaminating water systems^101–103^, and with forage cultivation under fertigation with Si, the P extracted from the soil would be continuously exported in the forage, without risk to the environment.

Thus, the third hypothesis of this research can be partially accepted, since fertigation with Si enabled to attenuate the excess P in both soils, but its benefits were not sufficient to mitigate P deficiency in the evaluated period. This reinforces in an unprecedented way that Si is more effective to attenuate P toxicity in relation to P deficiency in this species.

### Benefits of Si in forage plants under P sufficiency

Surprisingly, it was observed that in plants cultivated under P sufficiency, the supply of Si resulted in a change in stoichiometry, balancing the C:N and N:P ratios, especially in forages cultivated in Oxisol, also balancing the C:P ratio in Entisol in the second cut. Therefore, the potential of Si to increase nutritional efficiency in forages is unveiled due to greater stoichiometric homeostasis, especially of C:N and N:P. These changes in stoichiometry resulted in an increase in the use efficiency of P and N in the two forage cuts with sufficient P, associated with a decrease in the rate of senescent leaves and an increase in tillering. Consequently, there was an increase of approximately 5% in the dry matter production of plants cultivated in Oxisol. In Entisol, stoichiometric changes were restricted and were not sufficient to increase dry matter production in plants under sufficiency of P.

The role played by silicon as a facilitator of the balance of mineral nutrients in the plant and a mediator of morphological and biochemical changes^104,105^ may explain the increase in plant growth even without nutritional stress. Furthermore, high levels of Si in the shoots, such as those found in this study, favor the expression of the benefits of Si to plants, with recent studies reporting production gains due to stoichiometric balance and efficiency in the use of macronutrients such as N and P in different species cultivated without stress, such as forage plants cultivated in a hydroponic system^36,44^, sugarcane^37,106^, and sorghum ^107^.

These results have an important practical implication, as they allow indicating this beneficial element via fertigation for the cultivation of forage without P imbalances, especially if cultivated in Oxisol in an irrigated system.

## Conclusions and future perspective

Fertigation with Si nanoparticles promotes the absorption of the element by the forage, improves C:N:P homeostasis and the nutritional efficiency of plants cultivated under P deficiency and excess P, and reduces plant senescence regardless of P level, attenuating the stress caused especially by P toxicity in Entisol and in Oxisol, as well as in plants without nutritional stress grown in Oxisol.

The supply of Si increases the dry mass production of plants in the excessive state of P grown in Entisol, and also in plants grown under adequate and excessive states of P in Oxissol. Leaf senescence showed a lower value with addition of Si in plants grown in Oxisol in the three nutritional states of P.

The research revealed that using Si via fertigation triggers modifications in C:N:P stoichiometry that are relevant to the cultivation of *M. maximus* cv. Zuri, although the magnitude of the effects depends on the growth cycle, soil, and P nutritional status.

This study opens the way to expand research on the potential of Si via fertigation in forages of other species and also in other soils, having global implications in view of the occurrence of P deficiency and toxicity, which undermine the sustainability of forage cultivation in many countries.

## Data Availability

Datasets generated or analyzed during the present study are available from the corresponding author upon reasonable request.
